# Commentary on Population matched (pm) germline allelic variants of immunoglobulin (IG) loci: relevance in infectious diseases and vaccination studies in human populations

**DOI:** 10.1038/s41435-021-00152-6

**Published:** 2021-10-19

**Authors:** Andrew M. Collins, Ayelet Peres, Martin M. Corcoran, Corey T. Watson, Gur Yaari, William D. Lees, Mats Ohlin

**Affiliations:** 1 School of Biotechnology and Biomolecular Sciences, University of New South Wales, Sydney, NSW Australia; 2 Bioengineering, Faculty of Engineering, Bar Ilan University, Ramat Gan, Israel; 3 Bar Ilan Institute of Nanotechnologies and Advanced Materials, Bar Ilan University, Ramat Gan, Israel; 4 Department of Microbiology, Tumor and Cell Biology, Karolinska Institute, Stockholm, Sweden; 5 Department of Biochemistry and Molecular Genetics, University of Louisville School of Medicine, Louisville, KY USA; 6 Institute of Structural and Molecular Biology, Birkbeck College, University of London, London, UK; 7 Department of Immunotechnology, Lund University, Lund, Sweden

**Keywords:** Antibodies, Immunogenetics

**Dear Editor**,

In their recent publication, Khatri et al. [1] describe an immunoglobulin germline gene database inferred from short-read genomic sequence data derived from five superpopulations. The development of methods for the compilation of more complete and accurate germline gene databases would be an important achievement, but we do not believe that this has been achieved. Existing databases are clearly incomplete. Germline sequences differ substantially between subjects, and alleles found in some populations may be absent in others. Existing databases are likely biased towards alleles found in European populations and may lack many sequences found in understudied populations [2]. Improved, properly designed and curated germline gene databases are therefore needed for analysis of antibody repertoires.

Extensive efforts are under way to better document germline genes, and the study by Khatri et al. represents one such effort. Yet, their use of short-read sequencing data from the 1000 Genomes Project involves special challenges that we believe have not been met. The pitfalls of using short read data for genetic analysis of the IG loci have been discussed in detail previously [3–5]. Here, we focus on specific issues that call into question the completeness and accuracy of the pmIG database, and highlight shortcomings in the methodology which may explain them. We are motivated by concerns that less-informed users might be drawn towards the use of pmIG because it appears to contain a larger number of identified alleles than other databases. We believe that despite the breadth of the database, its problems compromise its utility. For brevity, the discussion is restricted here to the heavy chain IGHV genes, but the comments are equally relevant to the other immunoglobulin gene loci.

We first draw attention to the omission of many genes and alleles from the pmIG database. One reason for this is that the use of the GRCh37 assembly in analyses conducted to construct the pmIG database necessarily restricts it to those genes that are annotated in the assembly [3]. This excludes “duplicated” genes as noted by the authors, but also those present in other structurally variant haplotypes. In total, 15 known open reading frame IGHV genes are missing from GRCh37, and therefore from pmIG. These amount to approximately one fourth of all functional/ORF IGHV genes, and account for 43 alleles found in the IMGT and OGRDB databases. Importantly, the functional, missing genes have been commonly observed in many studies [5–12]. For example, the genes IGHV7-4-1, IGHV3-64D, IGHV5-10-1, IGHV4-30-2, and IGHV4-30-4, are identified in 46%, 65%, 62%, 75%, and 66%, respectively, of the transcribed repertoires for 421 human subjects curated by www.vdjbase.org [13].

Many common alleles of genes that are present in GRCh37 are also missing from pmIG, such as IGHV2-70*01 (found in 73% of subjects at VDJbase), IGHV3-11*06 (64%), and IGHV3-66*01 (53%), all of which are supported by genomic sequencing of unrearranged elements [8, 14, 15]. In the case of IGHV1-69, the absence of many well-documented alleles from pmIG can be traced to the absence of a single SNP in the pmIG results, as depicted in Khatri et al. Supplementary Fig. 2C [1]. These absences likely stem from the mis-mapping of reads to incorrect positions within the IGH locus, or their complete exclusion, with effects that carry through to variant calls found within the VCF files. Such cases can occur in duplicated and repetitive loci where it can be difficult to confidently assign short reads to a single position. In addition, reads from structural variants that differ from the reference assembly can also be mis-mapped to the closest-matching position within the reference, producing erroneous variant calls.

**Fig. 1:**
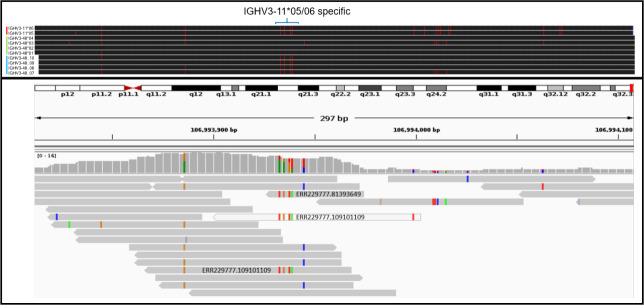
Problems caused by short read mis-assignments. Upper panel: Alignment of known and novel candidate germline sequences to GRCh37 reference assembly using the BLAT genome browser. Alleles IGHV3-11*05 and IGHV3-11*06 are indicated with the vertical red bar, IGHV3-48*01, IGHV3-48*02, IGHV3-48*03 and IGHV3-48*04 with the green bar and pmIG candidate alleles IGHV3-48_7, IGHV3-48_8, IGHV3-48_9 and IGHV3-48_10 with the blue bar. Position of the IGHV3-11 specific cluster of SNP variants is shown with the blue bracket. Lower panel: Assignment of low coverage sequences from 1000 genomes case HG00105, from the (HGO0105.mapped.ILLUMINA.bwa.GBR.low_coverage.20130415.bam) BAM file of GRCh37 assigned sequences visualized using the Broad Institute IVG viewer. Three short read sequences are incorrectly assigned to IGHV3-48 locus, ERR229777.81393649, ERR229777.109101109 and ERR229777.100808222 as shown by the presence of an IGHV3-11*05/06 specific segment containing four SNP variations, rs199879022 T (A in IGHV3-48), rs200437959 G (A in IGHV3-48) rs200973953 T (G in IGHV3-48) rs199815306 A (T in IGHV3-48). Four candidate IGHV3-48 germline sequences, IGHV3-48_7, IGHV3-48_8, IGHV3-48_9 and IGHV3-48_10, appear to be chimeric in origin, erroneously containing the IGHV3-11*05/06 segment.

Certain genes and alleles may be particularly susceptible to erroneous read mapping. The germline gene IGHV4-4 is often represented by the IGHV4-4*07 and IGHV4-4*02 alleles, but the variant IGHV4-4*01 is also common. IGHV4-4*07 is the allele present in the GRCh37 reference genome. All IGHV4-4 alleles in pmIG are close variants of IGHV4-4*07, strongly suggesting that reads derived from the IGHV4-4*01 and IGHV4-4*02 alleles are systematically misassigned, creating chimeric sequences that contribute either to the apparent diversity of the IGHV4-4*07-like variants, or to “novel” alleles of similar genes of the IGHV4 subgroup. Similarly, a short sequence string seen in half of the currently curated alleles of IGHV3-11 is not found in any of the IGHV3-11 alleles in pmIG, but rather appears in a similar sequence context in four pmIG alleles of IGHV3-48. Evidence for this mis-mapping can be observed in sample data from the 1000 Genomes Project (Fig. 1).

Identical mapping errors can be expected to occur in multiple datasets from the 1000 Genomes Project, as many individuals will share variant haplotypes. Misidentification of “novel” alleles can also be expected in these datasets, and multiple observations of a particular variant may point to systematic errors. We therefore do not accept the assertion of Khatri et al. [1] that there can be confidence in “novel alleles” that were identified in at least 7 haplotypes. The omission of numerous common alleles supported by various studies and methods, and in particular those identified by the amplification and sequencing of unrearranged genomic DNA, strongly suggests the presence of unexplained systematic errors. The authors also claim confidence in their methods based upon analysis of a single sample for which long-read sequencing was available [5]. They report the correct identification of 61 of 66 IGHV sequences in this sample but do not report whether false-positive calls were made. Khatri et al. explain their failure to find 5 of the 66 IGHV sequences as resulting from “differences in the human reference genome assembly used for mapping and calling alleles”. Although IGHV4-4*02, IGHV3-66*01, IGHV1-69*04, IGHV2-70*01 and IGHV2-70*15 are absent from GRCh37, this should not prevent their identification in a GRCh37-based analysis, as alleles of each of these genes are present in the reference assembly.

There are other anomalies in the database. For example, there is an extra base in framework region 3 in 4 of 5 alleles of IGHV3-49 and in 18 of 19 alleles of IGHV3-53. In addition, many (but not all) genes and alleles lack a number of bases at their 3’-end, an error that may compromise gene annotation and the analysis of the generation of diversity in the third hypervariable loop of the receptor. This anomaly is particularly surprising given that genomic sequences were used to generate the database. A third issue is that pmIG assigns novel allele names to sequences that only vary in their leader sequences. Multiple sequences may therefore contain V-exons that match a named allele in the IMGT database, creating challenges for any attempt to use a combined database for AIRR-seq analysis.

**Fig. 2:**
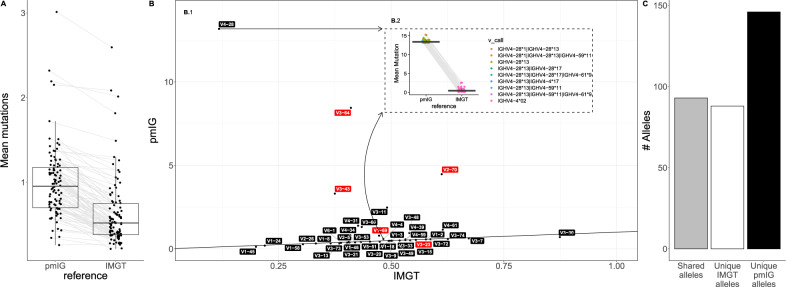
pmIG reference database introduces erroneous mutations. Repertoire analysis was performed on a naïve B-cell cohort (not expected to carry somatic mutations) of 98 individuals reported by Gidoni et al. (SRA: PRJEB26509) [6]. The repertoires were sequenced using the 5’RACE protocol and pre-processing was done as described in Gidoni et al. [6]. For the downstream analysis, repertoires were initially aligned (IgBLAST version 1.16.0) with the IMGT reference (March 29, 2021). Non-functional sequences, and functional sequences that were not full-length, were not assigned to a single V-gene unambiguously, or were assigned to a V-gene not present in the pmIG database were removed. The remaining 70% of functional sequences were then aligned with the pmIG reference database (downloaded from https://pmtrig.lumc.nl/ on July 15th, 2021) and the repertoires were compared. **A** For each repertoire, the mean mutation count was calculated using each reference database. Each dot represents the mean mutation count and each boxplot represents the variation within the cohort for each of the reference databases. **B** B.1 Each dot is the median of the mean individual mutation frequency per gene. The *X* axis is based on the IMGT reference and the *Y* axis is based on the pmIG reference. Red labels represent the genes with a duplicated copy in the chromosome (e.g., IGHV1-69/IGHV1-69D). B.2 Each dot represents an individual with IGHV4-4*02 in their IMGT data. These calls were matched via sequence IDs to calls in the matching pmIG datasets, and different gene annotations are shown with different colors. Where multiple allele calls were made, these calls are separated in the legend by vertical bars. The *X* axis shows the annotations to the two datasets. The *Y* axis shows the mean mutation numbers for the sequences assigned to the IGHV4-4*02 allele and to their matching calls in the pmIG dataset. **C** The count of alleles that are represented in the cohort for each of the reference databases.

Without additional work to explain and rectify errors and omissions of genes and alleles, the pmIG database is unsuited to many applications. Specifically, for AIRR-seq analysis, errors and omissions will result in erroneous germline gene and allele assignments, and ultimately impact the accuracy of other analyses including clonal inference and estimates of somatic hypermutation. The consequences will be most serious where absent genes and alleles are highly divergent from all sequences in the database. For example, if an AIRR-seq dataset from a donor carrying the allele IGHV4-30-2*01 (the most common allele of this gene) was analyzed using pmIG, reads derived from this gene would not be assigned to IGHV4-30-2, but instead to the closest germline sequence in pmIG, IGHV4-31_7, which differs by 13 nucleotides. To demonstrate the extent of these effects, we analyzed 98 naïve B cell repertoires. The cohort’s repertoires were aligned with the IMGT and pmIG reference sets, and mutation levels were compared across individuals and for individual genes. The calculated median mutation level across individuals was more than twice as high when sequences were aligned with pmIG compared to IMGT (Fig. 2A). This is a consequence of the many common genes and alleles present in this Norwegian cohort that are missing from pmIG (Fig. 2B). Comparison of the identified alleles using the two reference sets shows that 93 alleles were shared between the references, 88 were only observed in IMGT and 146 were observed only in pmIG (Fig. 2C).

The need to better document variation in the human immunoglobulin loci makes it important to exploit all available data sources where possible. At present however, in our view, the errors and omissions we have found suggest fundamental flaws in gene calling from short read data. We recognize that some and perhaps many alleles identified by Khatri and colleagues [1] are genuine, but we are presently unable to determine the reliability of any of the novel calls. If these calls were publicly linked to individual data sets, a more nuanced assessment of specific calls could be made, and inferences could be checked experimentally against their corresponding samples. Until such checks have been made, and until we better understand the challenges and limitations of short read data for the compilation of germline databases, we strongly advise against implementation of the current pmIG database, or similarly derived databases, in any AIRR-seq analysis.

When clear evidence is available that points to the reliability of adaptive immune receptor gene discovery from short-read sequencing projects, it will be important for processes to be established to review, name and document well-supported sequences. We believe this important task should be pursued through community-wide processes, in conjunction with naming authorities of the International Union of Immunological Societies. Such processes have been established for the evaluation of germline genes inferred from AIRR-Seq data [16, 17]. The substantial expansion of documented alleles that should result from short-read genome assemblies will then require more careful approaches than ever for the analysis of AIRR-Seq data. In particular, if alignments of mutated V(D)J sequences against the germline repertoire are to produce unequivocal alignments, the determination of individual genotypes prior to the reanalysis of AIRR-Seq datasets will need be recognized as an essential step in studies of the antibody repertoire.
